# Cardiopulmonary Resuscitation Knowledge Among Respiratory Therapy and Anesthesia Technology Students in Jordan: A Cross‐Sectional Study

**DOI:** 10.1155/tswj/1056717

**Published:** 2026-10-29

**Authors:** Eihab Khasawneh, Zaid I. Alkhatib, Alaa O. Oteir, Ragad Alkaraki

**Affiliations:** ^1^ Paramedic Program Department of Allied Medical Sciences Faculty of Applied Medical Sciences Jordan University of Science and Technology Irbid Jordan just.edu.jo; ^2^ Respiratory Therapy Program Department of Allied Medical Sciences Faculty of Applied Medical Sciences Jordan University of Science and Technology Irbid Jordan just.edu.jo

**Keywords:** anesthesia technology, bystander CPR, cardiopulmonary resuscitation, Jordan, respiratory therapy, training barriers

## Abstract

**Background:**

Cardiopulmonary resuscitation (CPR) knowledge among future critical‐care professionals is essential for improving survival after cardiac arrest. Evidence on CPR preparedness among respiratory therapy (RT) and anesthesia technology (AT) students in Jordan is limited. This study assessed CPR knowledge, willingness to perform CPR, and perceived motivators and barriers to CPR training.

**Methods:**

A cross‐sectional survey was conducted among RT and AT students (*n* = 360) in Jordan. Eligible participants were second‐year students and above. The questionnaire collected demographics, prior CPR training, a 10‐item CPR knowledge test (score 0–10), and self‐reported motivators and barriers. Descriptive statistics, *χ* ^2^ tests, and *t*‐tests were used.

**Results:**

Of the 360 participants, 46.9% had received CPR training; the mean age was 20.4 years, and 69.7% were female. The overall mean knowledge score was 5.22 (SD 2.00). Knowledge did not differ by prior training (5.27 vs. 5.18; *p* = 0.696). Students with basic life support training scored higher than those with advanced training (5.27 vs. 3.50; *p* = 0.001). Recognition of the emergency number (90.6%) and chest‐compression technique (66.9%) were the most correctly answered items, whereas compression depth (34.7%) and initial response steps (13.9%) showed the poorest knowledge. Almost all students (98.1%) were willing to attend CPR training. Key motivators were helping others (36.4%) and family cardiac disease (25.6%); barriers included lack of interest (34.2%), limited time (25.0%), and cost (19.4%).

**Conclusion:**

RT and AT students demonstrated moderate CPR knowledge but notable deficits in essential technical skills. High willingness to train supports integrating mandatory and recurrent CPR instruction into allied health curricula.

## 1. Introduction

Out‐of‐hospital cardiac arrest (OHCA) remains a leading global cause of death across all age groups [1]. Although cardiopulmonary resuscitation (CPR) has been established for decades as the cornerstone of cardiac‐arrest management, survival after OHCA remains low, ranging between 2% and 11% [1, 2]. More than half of such events are witnessed, yet fewer than 25% of victims receive bystander cardiopulmonary resuscitation (B‐CPR) [3, 4]. Early initiation of B‐CPR markedly improves outcomes, with survival rates up to fourfold higher among those receiving CPR before the arrival of emergency medical services [4–7]. Despite modest global improvement over the past four decades, survival in Western countries remains higher than in other regions, including the Middle East [2].

In Sweden, 30‐day survival after B‐CPR was 2.6 times higher than among those who did not receive CPR [6]. By contrast, a Jordanian study reported that more than two‐thirds of OHCA patients received no CPR before hospital arrival [8]. Consequently, national survival following OHCA is only 1.9%, far below the global average of about 10% [8, 9]. The rate nearly doubled (to 4.3%) when B‐CPR was performed [8].

Evidence consistently shows that CPR training increases willingness to perform resuscitation, thereby improving B‐CPR rates and OHCA survival [4, 10–12]. Similarly, in‐hospital cardiac arrest (IHCA) outcomes depend on early activation of the “chain of survival”—recognition, prompt high‐quality CPR, defibrillation, advanced resuscitation, postresuscitation care, and recovery and survivorship [13].

Healthcare professionals, particularly those in acute and critical‐care disciplines, are often first responders. Assessing their CPR competence and readiness is therefore crucial for both OHCA and IHCA outcomes.

In Jordan, survival following IHCA and OHCA remains poor (14.9% and 1.9%, respectively) [8, 14]. Limited CPR knowledge and training among the general population and healthcare providers are key contributing factors [9]. Previous Jordanian studies found CPR training rates of 14.5% among schoolteachers, 21.5% among allied health university students, and 29% among the public [10–12]. CPR knowledge was also found to be poor among the above‐mentioned groups of the Jordanian community [10–12]. However, these studies did not include students in respiratory therapy or anesthesia technology—disciplines whose graduates frequently manage cardiopulmonary emergencies and are expected to act promptly during cardiac arrest.

Accordingly, and since these students are expected to participate in recognition and management of prehospital and IHCA, this study is aimed at assessing CPR knowledge among students enrolled in Respiratory Therapy and Anesthesia Technology programs in Jordan, as well as their willingness to attend CPR training and perceived motivators and barriers to training. The findings are expected to inform the design of targeted educational interventions to enhance resuscitation preparedness in critical‐care contexts.

## 2. Methods

### 2.1. Study Design and Setting

This cross‐sectional questionnaire‐based study was conducted at the Faculty of Applied Medical Sciences, Jordan University of Science and Technology (JUST), Irbid, Jordan, during the academic year 2024/2025 (from September 2024 to June 2025). Reporting was guided by the Strengthening the Reporting of Observational Studies in Epidemiology (STROBE) checklist for cross‐sectional studies.

### 2.2. Participants

The study population comprised students enrolled in the Respiratory Therapy and Anesthesia Technology programs. Both are undergraduate BSc‐level allied health programs that prepare students for clinical roles involving airway, respiratory, anesthesia, and cardiopulmonary emergency care. Students in both programs receive CPR‐related theoretical and practical exposure through clinically oriented coursework and clinical training, including critical care for ATs and emergency respiratory and cardiac care for RTs. A nonprobability convenience sampling approach was used because, at the time of data collection, RT and AT programs in Jordan were offered exclusively at JUST. All eligible students present during scheduled teaching sessions were invited; first‐year students were excluded because they had not yet commenced clinical training. Participation was voluntary, and written informed consent was obtained from all participants.

### 2.3. Questionnaire

Data were collected using a structured, self‐administered questionnaire adapted from previously validated CPR questionnaires [12, 15]. The adapted instrument was reviewed for relevance, clarity, and face validity by the research team and CPR/clinical educators. The instrument consisted of three sections. The first section captured demographic and academic information, including sex, age, nationality, program, year of study, grade point average (GPA), prior CPR training, and previous CPR experience. The attitude questionnaire is reproduced in Appendix B.

The second section assessed adult CPR knowledge using 10 multiple‐choice questions covering recognition of cardiac arrest, the recommended compression‐ventilation ratio, compression depth and rate, and appropriate emergency actions. The items focused primarily on basic life support actions that are relevant to both out‐of‐hospital and IHCA before advanced resuscitation support is available. Each correct answer was scored as 1, and incorrect or “do not know” responses were scored as 0, yielding a total knowledge score ranging from 0 to 10. The knowledge questionnaire is provided in Appendix A.

The final section assessed factors influencing participation in CPR training. Motivators were (1) the desire to help others, (2) personal interest, (3) compulsory academic requirements, and (4) having a family member with heart disease. Barriers were (1) unawareness of training locations, (2) lack of time, (3) cost, (4) lack of interest, and (5) limited availability of courses. All items were recorded as binary (yes/no) responses.

### 2.4. Data Collection and Management

Questionnaires were distributed in paper format during classroom sessions and collected immediately upon completion by a research assistant. Responses were manually entered into Microsoft Excel by the research team, and a random 10% were double‐entered to detect and correct data‐entry errors. The cleaned dataset was then imported into Stata (Version 17, StataCorp, College Station, Texas, United States) for analysis. Double entry was used as a data‐quality procedure and was not intended to address potential systematic bias arising from the sampling approach.

### 2.5. Statistical Analysis

All analyses were performed using Stata Version 17. Continuous variables were summarized as means and standard deviations (SD), and categorical variables as frequencies and percentages. Pearson′s *χ* ^2^ tests were used for categorical variables and independent‐samples *t*‐tests for continuous variables. Knowledge items were expressed as the percentage of correct responses, and the total knowledge score was presented as the mean (SD). Differences in mean knowledge scores across sex, program, and prior training were examined using independent‐samples *t*‐tests. Where applicable, mean differences were reported with 95% confidence intervals (CIs), and effect sizes were reported using Cohen′s *d* for mean score comparisons. The normality of continuous variables was assessed visually and by distributional summaries; given the large sample size, *t*‐tests were considered appropriate for the main comparisons, although analyses involving the small advanced‐training subgroup were interpreted cautiously. No formal adjustment for multiple comparisons was applied because the analyses were exploratory and descriptive; however, the possibility of inflated Type I error due to multiple testing was considered when interpreting the findings. A *p* value < 0.05 was considered statistically significant.

### 2.6. Ethical Considerations

This study was conducted in accordance with the Helsinki Declaration. The study protocol was reviewed and approved by the Institutional Review Board of JUST. Participation was voluntary, and all responses remained anonymous and confidential (IRB No. AO‐20180064).

## 3. Results

### 3.1. Participants′ Demographics

Table 1 shows the demographic characteristics of the included participants with comparisons between CPR‐trained and untrained participants. A total of 360 eligible students were approached and included in the analysis, representing near‐complete coverage of the accessible RT and AT student population during the data collection period. Of these, 46.9% (*n* = 169) had previously received CPR training. The mean age of participants was 20.4 years (SD 0.8), with no significant difference between trained and untrained groups (20.3 vs. 20.5 years, *p* = 0.181). Females represented the majority of the cohort (251, 69.7%), whereas males accounted for 30.3% (*n* = 109); the distribution by sex did not differ significantly by training status (*p* = 0.060).

**Table 1:** Participants′ demographics with a comparison between CPR‐trained versus untrained participants.

Characteristic	Total (*n* = 360) (%)	Trained (*n* = 169) (%)	Untrained (*n* = 191) (%)	*p*
Age (mean [SD; 95% CIs], years)	20.4 (0.8; 20.3–20.5)	20.3 (0.8; 20.2–20.4)	20.5 (0.9; 20.4–20.6)	0.181^†^
Sex				0.060 ^∗^
Male	109 (30.3)	43 (39.4)	66 (60.6)	
Female	251 (69.7)	126 (50.2)	125 (49.8)	
Program (major)				< 0.001 ^∗^
Respiratory Therapy	178 (49.4)	137 (77.0)	41 (23.0)	
Anesthesia Technology	182 (50.6)	32 (17.6)	150 (82.4)	
Willing to attend CPR training				0.190 ^∗^
Yes	353 (98.1)	164 (46.5)	189 (53.5)	
No	7 (1.9)	5 (71.4)	2 (28.6)	
Ever performed CPR				< 0.001 ^∗^
Yes	35 (9.7)	29 (82.9)	6 (17.1)	
No	325 (90.3)	140 (43.1)	185 (56.9)	
GPA (mean [SD])	3.27 (0.51; 3.2–3.3)	3.36 (0.53; 3.3–3.4)	3.19 (0.49; 3.1–3.3)	0.001^†^

Abbreviations: 95% CIs, 95% confidence intervals; CPR, cardiopulmonary resuscitation; GPA, grade point average; SD, standard deviation.

^∗^Pearson′s *χ* ^2^ test.

^†^Independent‐samples *t*‐test.

Almost half of the participants were enrolled in the Respiratory Therapy program (178, 49.4%), and the remainder in Anesthesia Technology (182, 50.6%). Training status differed markedly between the two programs: 77.0% of respiratory therapy students had received CPR training compared with only 17.6% of Anesthesia Technology students (*p* < 0.001).

The vast majority of students (353, 98.1%) expressed willingness to attend CPR training, with no significant difference between groups (*p* = 0.190). One in 10 participants (35, 9.7%) reported having ever performed CPR, which was substantially more common among trained students than untrained (82.9% vs. 17.1%, *p* < 0.001). The mean GPA for the cohort was 3.27 (SD 0.51), with trained students achieving significantly higher GPAs than their untrained peers (3.36 vs. 3.19, *p* = 0.001).

### 3.2. Knowledge of CPR

The mean total knowledge score among participants was 5.22 (SD 2.00) out of a maximum of 10, corresponding to an average of 52.1% correct responses. Performance varied considerably across individual items (Table 2). The highest proportion of correct responses was observed for knowledge of the national emergency number (90.6%) and the correct chest compression technique (66.9%). Correct identification of the adult compression–ventilation ratio (61.4%) and recommended compression rate (60.6%) was also relatively common.

**Table 2:** Item‐level and overall knowledge of cardiopulmonary resuscitation (CPR) among students (*n* = 360).

Knowledge question	Correct answer (%)	Mean score (SD) ^∗^
Q1. You are alone and find an adult lying on the floor: What is the most important first step?	13.9	1.4 (0.35)
Q2. Which of the following is true regarding CPR?	57.2	5.7 (0.50)
Q3. What is the compression‐to‐breathing ratio for an adult?	61.4	6.1 (0.49)
Q4. What is the recommended number of compressions per minute for an adult?	60.6	6.1 (0.49)
Q5. Which of the following is a characteristic of effective CPR?	35.6	3.6 (0.48)
Q6. What is the recommended depth of chest compressions for an adult?	34.7	3.5 (0.48)
Q7. Once CPR is indicated, within what maximum time should chest compressions be initiated?	47.8	4.8 (0.50)
Q8. Which of the following describes an effective chest compression technique?	66.9	6.7 (0.47)
Q9. What is the emergency number in Jordan?	90.6	9.1 (0.29)
Q10. Does sudden loss of consciousness/collapse indicate a need for CPR?	53.6	5.4 (0.50)
Average across all items/total knowledge score	52.1	5.22 (2.00)

^∗^For item‐level rows, the mean score represents the proportion correct multiplied by 10 to place each item on the same 0–10 scale as the total knowledge score. Each item was coded 1 for *correct* and 0 for *incorrect*/“do not know.” CPR, cardiopulmonary resuscitation; Cronbach^′^s alpha for the 10 − item scale = 0.53 (*N* = 360).

In contrast, knowledge of the most appropriate first step when encountering an unresponsive adult was very limited, with only 13.9% (*n* = 50) of students answering correctly. Similarly, less than 40% of respondents identified the correct depth of chest compressions (34.7%) or the key characteristics of effective CPR (35.6%). Approximately half of the students recognized that sudden collapse indicates a need for CPR (53.6%) or the maximum recommended time before initiating compressions (47.8%).

Internal consistency of the 10‐item scale was modest (Cronbach^′^s *α* = 0.53).

Mean knowledge scores did not differ significantly by sex, program, or previous CPR training (Table 3). Male students had a slightly higher mean score than females, but the difference was not statistically significant (5.42 vs. 5.14; mean difference 0.28; *p* = 0.213). Scores were also similar between respiratory therapy and anesthesia technology students (5.21 vs. 5.24; mean difference −0.03; *p* = 0.893), and between students who had received prior CPR training and those who had not (5.27 vs. 5.18; mean difference 0.09; *p* = 0.696). However, knowledge scores varied significantly by the level of CPR training. Students who reported basic training scored higher than those with advanced training (5.27 vs. 3.50; mean difference 1.77; *p* = 0.001).

**Table 3:** Knowledge scores by sex, program, prior CPR training, and level of CPR training (*n* = 360).

Characteristic	*n*(%)	Mean (SD)	95% CI	*p* ^∗^
Sex				0.213
Male	109 (30.3)	5.42 (2.07)	5.0–5.8	
Female	251 (69.7)	5.14 (1.98)	4.9–5.4	
Program (major)				0.893
Respiratory therapy	178 (49.4)	5.21 (2.16)	4.9–5.5	
Anesthesia technology	182 (50.6)	5.24 (1.84)	5.0–5.5	
Previous CPR training				0.696
Yes	169 (46.9)	5.27 (1.95)	5.0–5.6	
No	191 (53.1)	5.18 (2.06)	4.9–5.5	
Level of CPR training				0.001^†^
Basic	168 (92.3)	5.27 (1.94)	5.0–5.6	
Advanced	14 (7.7)	3.50 (1.95)	2.4–4.6	

Abbreviations: CI, confidence interval; CPR, cardiopulmonary resuscitation; SD, standard deviation.

^∗^Independent‐samples *t*‐tests.

^†^The advanced‐training subgroup was small (*n* = 14); this comparison should be interpreted cautiously.

After adjustment for major and CPR training, GPA was not significantly associated with knowledge score (adjusted *B* = 0.070, 95% CI: −0.824 to 0.964, *p* = 0.879).

Major was not significantly associated with knowledge score after adjustment for GPA and CPR training (adjusted *B* = −0.549, 95% CI: −1.670 to 0.572, *p* = 0.338). Similarly, CPR training was not significantly associated with knowledge score (adjusted *B* = −0.890, 95% CI: −2.007 to 0.227, *p* = 0.120).

### 3.3. Factors Influencing Participation in CPR Training

Students identified several factors that would encourage them to attend CPR training (Figure 1). The most frequently reported motivator was the desire to help others (36.4%). Having a family member with heart disease (25.6%) and compulsory university requirements (20.8%) were also commonly reported. In contrast, only a small proportion (3.9%) indicated personal interest alone as a motivator.

**Figure 1:**
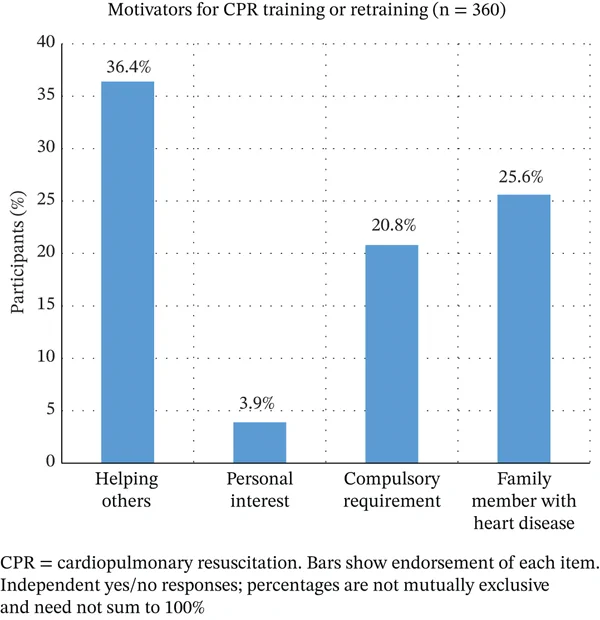
Reported motivators to CPR training or retraining among students (*n* = 360). Items were recorded as independent binary endorsements; therefore, percentages are not mutually exclusive and do not sum to 100%.

Reported barriers to attending CPR training are shown in Figure 2. The most common barrier was lack of interest (34.2%), followed by lack of time (25.0%) and the cost of training (19.4%). Lack of course availability was reported by 12.8% of students, whereas unawareness of training locations was rarely mentioned (2.5%).

**Figure 2:**
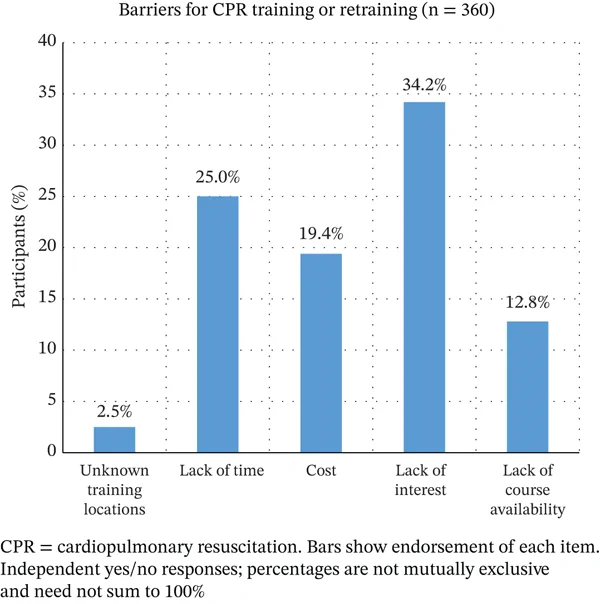
Reported barriers to CPR training or retraining among students (*n* = 360). Items were recorded as independent binary endorsements; therefore, percentages are not mutually exclusive and do not sum to 100%.

## 4. Discussion

To our knowledge, this is the first study to assess CPR knowledge among RT and AT students in Jordan. This study revealed that although nearly half of the students had previously received CPR training, their overall knowledge remained inadequate.

A study in Jordan, including 883 allied health profession students, reported a low overall CPR knowledge mean score of 3.9 ± 1.7 out of 10 points, with all majors answering less than 50% of the questions correctly [12]. Findings from our study indicate a higher level of knowledge than that of other allied health profession students in Jordan. A possible reason for this difference is that RTs and ATs received more CPR training (46.9%) than allied health profession students (21.5%). Another Jordanian study was conducted using a quasi‐experimental design and involved 510 allied health students who showed significant improvement in knowledge, attitude and practice of CPR after providing educational intervention programs [16]. Allied health students are future professionals and an integral part of the healthcare system. Yet CPR courses are not adequately offered in many programs, possibly because they are perceived as irrelevant to their scope of practice in healthcare settings. Collective efforts across programs should raise awareness of CPR and require it as a mandatory component in all allied health curricula.

Regional studies conducted in Saudi Arabia among university students reported varying levels of CPR knowledge. Although several studies showed unsatisfactory knowledge of CPR [17–21], a recent study at Najran University showed a moderate level of knowledge among undergraduate medical students, achieving a mean knowledge score of 6.34 ± 2.17 out of 10 [22]. These findings are consistent with our study results and other local research among allied health students in Jordan, indicating that insufficient preparedness and generally low CPR knowledge remain regional concerns, despite the slight progress reported elsewhere.

Only 34.7% of students in our study correctly identified the recommended depth of chest compressions, and 35.6% recognized characteristics of effective CPR. These proportions were lower than those reported at Najran University, where 43.4% and 65.1% of students correctly answered the recommended depth of chest compressions and vital CPR characteristics, respectively [22]. The proportion correctly identifying the recommended compression depth was also markedly lower than reported in other regions. A study conducted across Europe in 2019 involved 1012 medical students, of whom 69.7% correctly identified compression depth [23]. This demonstrated a knowledge deficiency in particular technical CPR skills, emphasizing the need to integrate structured training and hands‐on skill practice within university curricula.

Our study found that CPR knowledge did not differ significantly by sex, consistent with an existing study among 883 allied health students in Jordan, the majority of whom were female (73.1%), with no significant gender differences in CPR knowledge [12]. Nevertheless, previous research has yielded conflicting findings regarding the association between gender and CPR knowledge. In a study of 823 Egyptian medical students, 88.3% of whom were male, CPR knowledge scores were significantly higher among females than among males [24]. In contrast, among 258 medical students in Saudi Arabia, 55% of whom were male, males demonstrated a significantly higher level of knowledge than females [22]. Given the findings of prior studies, direct comparisons between them should be interpreted cautiously due to differences in sample size, gender distribution, and assessment tools. Several factors may also influence the observed gender differences in CPR knowledge, including variations in curricula, teaching methods, and cultural factors. In Jordanian culture, the absence of gender differences may reflect equal access to CPR training and education, as well as academic norms that expect similar performance from both genders.

Additionally, trained students had insufficient knowledge despite prior CPR training, with no significant difference in knowledge scores compared with untrained students. Findings from allied medical students in Jordan revealed a higher mean score among trained students (4.6 ± 1.6) than among untrained students (3.8 ± 1.6); however, overall knowledge levels remained poor in both groups [12]. Similarly, Abbas et al. [25] reported that among 250 medical students in Pakistan, those who had received training achieved higher mean CPR knowledge scores compared to their untrained peers; but overall performance remained below 50%, highlighting the need for annual reinforcement. Our findings may be attributed to the retention of theoretical knowledge of CPR, which can decline within 1–6 months after completing BLS training, as reported in previous studies among healthcare and nursing students, emphasizing the need for continuous refresher courses [26–28].

We found that students who had completed BLS courses achieved higher knowledge scores than those who had completed advanced life support courses. This finding may be attributed to the difference in content focus between the two courses. BLS training emphasizes the core principles of single‐rescuer CPR, such as chest‐compression depth, rate, and sequence, which were primarily assessed in this study.

In contrast, ALS courses typically prioritize rhythm recognition, pharmacological management, and team‐based interventions, assuming prior mastery of BLS skills. Consequently, ALS participants may have had less recent reinforcement of fundamental CPR knowledge. However, this finding should be interpreted cautiously because only 14 students reported advanced training, which may have produced unstable estimates despite the relatively large observed effect size.

Regardless of their training status, 98.1% of students in both majors expressed willingness to attend CPR training or retraining, primarily motivated by the desire to help people (36.4%). Similar results were found in research involving allied health university students in Jordan, who demonstrated willingness to attend CPR training, with helping others (44.1%) as the leading motivator [12]. The second leading motivator was having family members with cardiac diseases, which may indicate a sense of responsibility to be prepared in the event of a cardiac arrest. It is worth noting that cardiovascular diseases are the leading cause of death in Jordan, responsible for 39% of all deaths [29]. Research on training family members of high‐risk patients in CPR remains limited. However, existing research demonstrated that CPR skills can be effectively taught to family members of cardiac patients and suggests hospitalization as a critical setting for educating families in CPR [30–33]. A systematic review showed that targeted BLS training for family members of high‐risk cardiac patients improved BLS competency, increased willingness to act, and reduced anxiety [34]. Therefore, family members of high‐risk cardiovascular patients could serve as a targeted community for policymakers and healthcare sectors to raise awareness and provide BLS courses.

Although participants reported various motivators for engaging in CPR training, several barriers were also identified. The most frequent barrier in our study was lack of interest, reported by 34.2% of students, followed by lack of time (25%) and the financial cost of training (19.4%).

A lack of interest may be attributed to perceived irrelevance, limited awareness of CPR benefits, or anxiety about skill performance. Time constraints and the high financial cost of courses were commonly reported across previous studies [12, 35, 36]. These barriers were addressed by the American Heart Association (AHA), which is aimed at increasing the accessibility of CPR courses [13]. Various methods have been developed, including hands‐only CPR for laypersons and instructional videos for self‐learning [37].

### 4.1. Recommendations and Limitations

Based on the findings of this study, CPR training should be integrated as a mandatory and recurrent component in undergraduate allied health curricula, with particular emphasis on practical CPR skills, technical elements such as compression depth and rate, and refresher training to prevent knowledge decay. This is especially relevant because respiratory therapy and anesthesia technology are relatively new programs in Jordan and were established in response to the increased demand for specialized healthcare professionals following the COVID‐19 pandemic within the Allied Health Sciences department at JUST. Furthermore, research should evaluate the effectiveness of different teaching methods, such as simulation‐based scenarios, peer‐assisted training, and video‐based instruction, in improving both short‐term knowledge retention and long‐term practical CPR performance. Future studies should also investigate willingness and attitudes toward CPR training among family members of high‐risk cardiovascular patients. The recommendation for recurrent training is consistent with the 2025 AHA resuscitation education guidelines, which recommend booster sessions after massed training and consider spaced learning a reasonable alternative [38].

Several limitations should be acknowledged. First, the cross‐sectional design captures students′ knowledge at a single point in time and does not allow assessment of changes following training or progression through the program. Second, although the study achieved near‐complete coverage of the accessible RT and AT student population at JUST, the use of nonprobability convenience sampling may limit representativeness and introduce selection bias. Third, the single‐center setting restricts generalizability to students in other institutions or regions.

Fourth, CPR competence includes both theoretical knowledge and psychomotor skill performance; therefore, assessing knowledge through a questionnaire alone may not reflect students′ ability to perform high‐quality CPR in real clinical situations. Fifth, the modest internal consistency of the 10‐item knowledge scale (Cronbach^′^ *s* *α* = 0.53) suggests that the total score should be interpreted cautiously as a single unified construct. Sixth, multiple statistical comparisons were conducted without formal multiplicity correction, which may increase the risk of Type I error. Finally, subgroup analyses involving students with advanced training should be interpreted cautiously because of the small number of participants in this group. Future studies should consider multicenter recruitment, probabilistic sampling, longitudinal designs, objective CPR skill assessment using simulation or manikins, and adjusted analyses using individual‐level data to better evaluate determinants of CPR knowledge and competence.

## 5. Conclusion

RT and AT students demonstrated a strong willingness to attend CPR training and retraining, but their CPR knowledge was suboptimal, particularly for essential technical elements of high‐quality CPR. These findings support introducing mandatory and recurrent CPR training within allied health curricula and reinforcing knowledge through regular refresher sessions. Future research should evaluate teaching methods such as simulation‐based and video‐based instruction to improve both knowledge retention and practical CPR skill application.

## Author Contributions

E.K.: data curation, validation, formal analysis, methodology, project administration, resources, and writing original draft. Z.I.A.: conceptualization, data collection, data curation, validation, formal analysis, methodology, resources, and writing original draft. A.O.O.: formal analysis, methodology, validation, and writing—review and editing. R.A.: writing—review and editing.

## Funding

No funding was received for this manuscript.

## Conflicts of Interest

The authors declare no conflicts of interest.

## Data Availability

The data that support the findings of this study are available from the corresponding author upon reasonable request due to privacy reasons.

## Appendix 1

**Table A1:** Knowledge questionnaire.

Knowledge questions	Answers
You were alone and sighted an adult laying on the floor, what would be the most important step to do?	Check consciousness and breathing.
	Check pulse.
	Start compressions immediately.
	Call for help or emergency number.

Which of the following is true regarding CPR?	CPR starts with chest compressions.
	CPR starts with mouth to mouth ventilation.
	CPR starts with mouth to mouth ventilation and chest compressions simultaneously.
	Giving a mouth‐to‐mouth ventilation is more important and superior to chest compression.

What is the compressions to ventilation ratio for an adult patient?	30 compression:2 ventilations.
	30 compression:5 ventilations.
	5 compression:1 ventilation.
	15 compression:1 ventilation.

What is the number of compressions per minute for an adult patient?	100–120 compressions per minute.
	More than 120 compressions per minute.
	80–100 compressions per minute.
	60–80 compressions per minute.

Which of the following is a characteristic of true effective CPR?	Allowing full chest recoil after each compression.
	Compression without allowing chest recoil.
	Compressing fast but not hard.
	Compressing slowly.

What is the depth of compression for an adult patient?	5–6 cm.
	2–3 cm.
	3–4 cm.
	At least 6 cm.

Once confirmed the need for CPR, chest compressions should start within a maximum of.	10 s.
	5 s.
	15 s.
	30 s.

Which of the following is a characteristic of true effective CPR?	Pushing (compressing) hard and fast.
	Pushing (compressing) with medium speed.
	Pushing (compressing) slowly.
	Pushing (compressing) with medium power.

What is the emergency number in Jordan?	911.
	000.
	119.
	112.

Sudden loss of consciousness/collapse may indicate a need for CPR.	Yes.
	No.

## Appendix 2

**Table A2:** Attitude questionnaire.

Questionnaire items	Strongly agree	Agree	Neutral	Disagree	Strongly disagree
I would provide MMV for strangers.	5	4	3	2	1
I would provide MMV for relatives.	5	4	3	2	1
I would provide MMV for the other gender.	5	4	3	2	1
I avoid providing MMV due to fear of infection^a^.	5	4	3	2	1
I would provide MMV for children.	5	4	3	2	1
I avoid providing CC due for fear of infection^a^.	5	4	3	2	1
I would provide CC for relatives.	5	4	3	2	1
I would provide CC for strangers.	5	4	3	2	1
I believe that CPR is harmful^a^.	5	4	3	2	1
I would like to learn and practice CPR encouraged by cultural values and religious beliefs.	5	4	3	2	1
I believe that CPR is important and can increase the patients′ survival.	5	4	3	2	1

Abbreviations: CC, chest compressions; CPR, cardiopulmonary resuscitation; MMV, mouth‐to‐mouth ventilation.

^a^Inverse scoring.
